# Ventral Striatal Dopamine Synthesis Capacity Predicts Financial Extravagance in Parkinson’s Disease

**DOI:** 10.3389/fpsyg.2013.00090

**Published:** 2013-02-27

**Authors:** Andrew D. Lawrence, David J. Brooks, Alan L. Whone

**Affiliations:** ^1^School of Psychology, Cardiff UniversityCardiff, UK; ^2^Centre for Neuroscience, Department of Medicine, Imperial College LondonLondon, UK; ^3^Department of Neurology, Frenchay HospitalBristol, UK

**Keywords:** dopa decarboxylase, dopamine, disordered gambling, externalizing, impulse control disorders, impulsivity, reward, ventral striatum

## Abstract

Impulse control disorders (ICDs), including disordered gambling, can occur in a significant number of patients with Parkinson’s disease (PD) receiving dopaminergic therapy. The neurobiology underlying susceptibility to such problems is unclear, but risk likely results from an interaction between dopaminergic medication and a pre-existing trait vulnerability. Impulse control and addictive disorders form part of a broader psychopathological spectrum of disorders, which share a common underlying genetic vulnerability, referred to as externalizing. The broad externalizing risk factor is a continuously varying trait reflecting vulnerability to various impulse control problems, manifested at the overt level by disinhibitory symptoms and at the personality level by antecedent traits such as impulsivity and novelty/sensation seeking. Trait “disinhibition” is thus a core endophenotype of ICDs, and a key target for neurobiological investigation. The ventral striatal dopamine system has been hypothesized to underlie individual variation in behavioral disinhibition. Here, we examined whether individual differences in ventral striatal dopamine synthesis capacity predicted individual variation in disinhibitory temperament traits in individuals with PD. Eighteen early-stage male PD patients underwent 6-[^18^F]Fluoro-l-DOPA (FDOPA) positron emission tomography scanning to measure striatal dopamine synthesis capacity, and completed a measure of disinhibited personality. Consistent with our predictions, we found that levels of ventral, but not dorsal, striatal dopamine synthesis capacity predicted disinhibited personality, particularly a propensity for financial extravagance. Our results are consistent with recent preclinical models of vulnerability to behavioral disinhibition and addiction proneness, and provide novel insights into the neurobiology of potential vulnerability to impulse control problems in PD and other disorders.

## Introduction

Several addictive and impulse control disorders (ICDs) have been associated with Parkinson’s disease (PD) and its treatment with dopaminergic medication, including disordered gambling (Gallagher et al., 2007), substance dependence (Bienfait et al., 2010), and the addiction-like excessive use of dopaminergic medications, or DA dysregulation syndrome (Lawrence et al., 2003). The prevalence of ICDs in medicated PD patients was estimated at ∼14% in a large (*n* > 3000) multicentre study, with >25% of affected individuals having multiple ICDs (Weintraub et al., 2010). The development of ICDs in PD likely results from an interaction between dopaminergic medication and an underlying vulnerability, rather than from PD itself, since: (a) only a (substantial) minority of medicated PD patients develop ICDs (Weintraub et al., 2010); (b) ICDs are no more frequent in patients with *de novo* PD than in the general population (Weintraub et al., 2013); (c) ICDs can develop in non-PD individuals treated with dopaminergic medication (O’Sullivan et al., 2010; Voon et al., 2011a); and (d) a family history of gambling problems is a risk factor for the development of dopaminergic medication-linked ICDs (Weintraub et al., 2010; Voon et al., 2011a).

This precursive ICD vulnerability likely reflects variation in pre-existing temperament/personality (Dagher and Robbins, 2009), in particular, variation in the broad temperament dimension of trait disinhibition (vs. constraint), encompassing impulsivity, novelty/sensation seeking, non-planning, low self-control, and related constructs (Markon et al., 2005). In PD, just as in non-PD populations (Sher and Trull, 1994; MacLaren et al., 2011), phenotypic associations between trait disinhibition and substance use and ICDs (and their comorbidity) have been repeatedly demonstrated (Evans et al., 2005; Voon et al., 2011b).

These findings suggest etiologic similarities between ICDs and addictions in PD and the broader domain of externalizing (EXT) psychopathology in non-PD populations (Krueger et al., 2007). Patterns of phenotypic, environmental, and genetic relations among DSM-defined substance misuse and antisocial disorders are best accounted for by models positing a shared broad EXT factor or vulnerability (Krueger et al., 2007). I.e., putatively distinct disorders are better understood as variants within a broader EXT spectrum of disorders, and the reason they systematically co-occur is because they share a common underlying genetic vulnerability (Krueger et al., 2007). This broad EXT vulnerability is a continuously distributed dimension of risk (Krueger et al., 2007). Hierarchical models of the EXT spectrum (Krueger et al., 2007) posit a general factor linking all EXT syndromes, as well as distinct etiological factors that differentiate among distinct EXT syndromes. Disordered gambling, frequently comorbid with substance abuse and antisocial personality disorder (Kessler et al., 2008) is also considered one variant of EXT (Oleski et al., 2011; Blanco et al., 2012; Forbush and Watson, 2013; but see Slutske et al., 2013).

A shared genetic diathesis underlies the phenotypic associations between EXT disorders and disinhibited personality traits, including novelty seeking (NS; Young et al., 2000; Agrawal et al., 2004). Furthermore, prospective studies suggest that high levels of these traits in childhood and adolescence predate and predict the emergence of EXT psychopathology in adulthood (Sher et al., 2000; Slutske et al., 2012). I.e., the temperamental antecedent of disinhibition provides the core endophenotype of EXT disorders (Clark, 2005), mediating their systematic co-variation (Khan et al., 2005). Hence, understanding the genetics and neurobiology of disinhibitory personality traits is critical to understanding EXT psychopathology, including its manifestation in the context of PD and its treatment with DA replacement therapies.

The DA system has frequently been hypothesized to underlie individual variation in trait disinhibition. According to one prominent model (Pickering and Gray, 1999), individual differences along this temperament dimension are argued to reflect variation in the reactivity of a neural behavioral activation system (BAS), centered on the ventral striatum (VS), and its dopaminergic irrigation, activated by cues for reward. Similarly, according to Cloninger (1987), the disinhibitory temperament trait of NS reflects genetically determined variation in dopaminergically mediated BAS reactivity. When activated, the BAS can be characterized as an impulsive “go” system that activates on-going appetitive behavior (Pine et al., 2010).

Research is accumulating to suggest that variation in the DA synthesis pathway in particular plays a key role in the etiology of EXT. DA synthesis occurs within DA neurons. Tyrosine is transported into the cell via amino acid carriers in the blood-brain barrier and cell membranes. Once in the intracellular space it is hydroxylated to l-3,4-dihydroxiphenylalanine (l-DOPA) by tyrosine hydroxylase (TH). l-DOPA is then decarboxylated by aromatic l-amino acid decarboxylase [AADC; also known as dopa decarboxylase (DDC)] to DA (Elsworth and Roth, 2009). Variants [single nucleotide polymorphisms (SNPs)] in the DDC gene have been associated with nicotine (Ma et al., 2005; Yu et al., 2006; Zhang et al., 2006), alcohol (Agrawal et al., 2011; Kristjansson et al., 2012) and illicit drug (Hack et al., 2011) misuse, and most recently, disordered gambling (Lind et al., 2012). Importantly, Derringer et al. (2010) found that a combination of multiple SNPs in the DDC gene predicted individual variation in sensation seeking traits, suggesting that genetic variation in DA synthesis is related to broad EXT risk.

Positron Emission Tomography (PET) can be used to study the activity of AADC/DDC in pre-synaptic DA terminals in the living brain. The PET tracer 6-[^18^F]fluoro-l-DOPA (FDOPA), a radioactive analog of l-DOPA, the precursor of DA, is taken up by pre-synaptic dopaminergic neurons and is metabolized by AADC/DDC to 18F-DA, which is trapped and stored within vesicles in the nerve terminals (Kumakura and Cumming, 2009). FDOPA uptake, quantified as the influx constant *K*_I_, can be used as a measure of AADC/DDC activity and vesicular storage capacity (Brown et al., 1999). High values for FDOPA *K*_I_ are observed in areas of dense DA nerve terminal innervation, such as the striatum, and FDOPA has been extensively used to probe the functional integrity of striatal dopaminergic neurons in PD (Brooks, 2010) where uptake correlates with the number of surviving nigrostriatal cell numbers (Snow et al., 1993). Notably, FDOPA studies in non-PD populations have shown relatively increased striatal *K*_I_ values in alcohol (Tiihonen et al., 1998) and nicotine (Salokangas et al., 2000) dependent individuals, although those early studies did not measure disinhibitory personality traits, and could not resolve dorsal and ventral striatal regions. Further, Laakso et al. (2005) found increased striatal FDOPA uptake in A1+ allele carriers of the Taq1A polymorphism of the *TTC12-ANKK1-DRD2* gene cluster, variation in which has been linked with EXT risk (Ducci et al., 2011).

Collectively, the research outlined above suggests that individual variation in DA synthesis capacity, particularly in the VS, underpins individual variation in trait disinhibition. Here, we tested the hypothesis that increased FDOPA uptake in VS, which is relatively spared in PD (Kish et al., 1988), would predict increased trait disinhibition or general EXT risk, in the context of PD.

## Materials and Methods

### Patients

We studied 18 men with early-stage (symptom duration <2 years) idiopathic PD based on UK PD Society Brain Bank diagnostic criteria. Table 1 summarizes their clinical characteristics. Disease severity was rated with the Unified Parkinson’s Disease Rating Scale (UPDRS) motor subscale while in an “off” condition withdrawn from medication before PET. Assessment of trait disinhibition, as well as PET scanning, was performed in a practically defined off state. Patients with pronounced tremor that would have produced difficulty with PET imaging, as well as patients with comorbid psychiatric or systemic physical illness were excluded. This cohort of 18 patients was drawn from a larger group of 186 PD patients that formed the multicentre REAL-PET investigation enrolled population. Drug escalation, tablet frequency, dose, and UPDRS motor response to medication have been described previously (Whone et al., 2003b). At the time of PET scanning, patients had only been started on dopaminergic medication within the previous 4–12 weeks, and were receiving low doses of either l-DOPA (between 300 mg to a maximum daily dose of ∼400 mg at time of PET) or a DA agonist (between 3 mg of ropinirole to a maximum daily dose of ∼8 mg at time of PET). Patients were administered the 30-item Geriatric Depression Scale (GDS), validated for use in PD (Ertan et al., 2005).

**Table 1:** **Patient characteristics**.

	Parkinson’s disease patients (*n* = 18)
Gender (male/female)	18/0
Age (years, mean ± SD, range)	64 ± 7, range 47–76
UPDRS motor in “off” (mean ± SD)	14.6 ± 6.5
GDS Depression (mean ± SD)	9.8 ± 8.9

The study was limited to men for several reasons. Firstly, ICDs and addictions in PD are more prevalent in males than females (Weintraub et al., 2010), as they are in non-PD populations (Eaton et al., 2012). Secondly, there are sex differences in DA synthesis capacity (Laakso et al., 2002). Thirdly, genetic factors for NS are excellent markers for EXT tendencies in males, but not females (Agrawal et al., 2004; Pitzer et al., 2007), although it is unclear whether this sex difference is an artifact of the manner in which males and females interpret the NS construct or whether there are true differences in the magnitude of genetic influence across males and females. It will be important in future studies to include females with PD.

Permission to undertake the study was granted by the Ethics Committee of Hammersmith, Queen Charlotte’s & Chelsea, and Acton Hospital Trust and all participants gave written informed consent following a full explanation of the procedure, in accordance with the declaration of Helsinki. The Administration of Radioactive Substances Advisory Committee (ARSAC) of the UK approved radioisotope use.

### Measurement of trait disinhibition (vs. constraint)

Our measure of trait disinhibition (vs. constraint) was based on NS from Cloninger’s Tri-dimensional Personality Questionnaire (TPQ; Cloninger, 1987). The version of the TPQ used here was a 100-item, self-administered, true-false instrument. The questionnaire is scored so that higher scores reflect greater NS.

As originally constructed (Cloninger, 1987) TPQ-NS comprised four narrow facet-level scales: Exploratory Excitability vs. Stoic Rigidity (NS1), Impulsiveness vs. Reflection (NS2), Extravagance vs. Reserve (NS3), and Disorderliness vs. Regimentation (NS4). When Ando et al. (2004), however, examined the genetic and environmental factor structure of NS, factor analysis of the genetic inter-correlations yielded factors that did not fully resemble the phenotypic structure of NS as proposed by Cloninger (1987). NS was revised (r-NS) to consist of Impulsiveness vs. Reflection (NS2), Extravagance vs. Reserve (NS3) and Disorderliness vs. Regimentation (NS4), excluding Exploratory Excitability vs. Stoic Rigidity (NS1). Further, Flory et al. (2006), using factor analysis in a large normative sample of middle-aged adults, found Impulsiveness vs. Reflection (NS2) and Extravagance vs. Reserve (NS3) to have high loadings on a “non-planning impulsivity” factor, together with the Barratt Impulsiveness Scale (BIS), whereas Exploratory Excitability vs. Stoic Rigidity (NS1) loaded on a distinct “Openness to Experience” factor, and Disorderliness vs. Regimentation (NS4) failed to load strongly on any single factor. Hence, in the current study, we focused on those r-NS facets most strongly linked to trait disinhibition: *Impulsiveness* (vs. Reflection; NS2; eight items) and *Extravagance* (vs. Reserve; NS3; seven items). Sample items include “*I often react so strongly to unexpected news that I say or do things that I regret,”* “*I often have to change my decisions because I had a wrong hunch or mistaken first impression”* (Impulsivity, NS2) and “*I often spend money until I run out of cash or get into debt from using too much credit,”* “*Because I so often spend too much money on impulse, it is hard for me to save money – even for special plans like a vacation”* (Extravagance, NS3).

### PET scanning protocol

Patients stopped medication at least 12 h prior to scanning. All participants underwent three-dimensional FDOPA PET using an ECAT EXACT HR++ (CTI/Siemens 966) camera, which covers an axial field of view of 23.4 cm and provides 95 transaxial planes. The tomograph has a spatial resolution of 4.8 + 0.2 mm FWHM (transaxial, 1 cm off axis) and 5.6 + 0.5 mm (axial, on axis) after image reconstruction (Spinks et al., 2000). A transmission scan, which corrects for attenuation of emitted radiation by skull and tissues, was acquired using a single rotating photon point source of 150 MBq of ^137^Cs. Thirty seconds after the start of the emission scan, 110 MBq of FDOPA in 10 ml normal saline was infused intravenously over 30 s. Three-dimensional sinograms of emission data were then acquired over 94 min as 26 time frames. Patients were placed in the scanner, orientated parallel to the orbito-metal line and head positioning was monitored throughout the scan.

### Image quantification

Parametric images of specific FDOPA uptake (K_I_ maps) were created at a voxel level for the whole brain using the Patlak graphical approach (Patlak and Blasberg, 1985) with a cerebellar cortex reference input function (Moore et al., 2003). Qualitative summated images created from the dynamic FDOPA time series by integrating all 26 frames of the dynamic image were also produced and then transformed into standard stereotaxic [Montreal Neurological Institute (MNI)] space using an FDOPA template created in-house from a healthy volunteer database. These so-called ADD images contain both tracer delivery and specific uptake information and provide adequate anatomical detail to allow stereotaxic manipulations. Subsequently, the K_I_ maps were also individually normalized to MNI stereotaxic space by applying the transformation parameters defined during the normalization of their respective ADD image. This spatial transformation of parametric images made it possible to perform a region of interest (ROI) analysis as described below.

### Region of interest analysis

**Figure 1:**
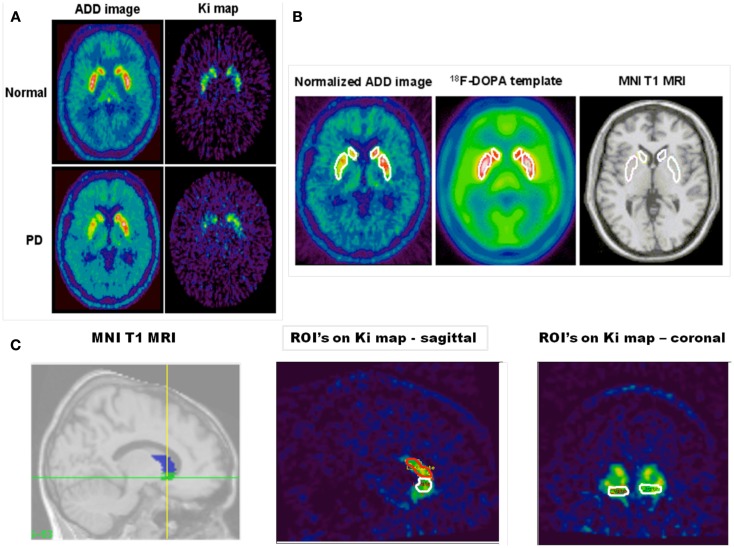
**The figure shows in (A) an FDOPA ADD (summed) and net influx rate constant (*K*_I_) image of a single slice from a healthy individual (top panels) and a patient with PD (bottom panels)**. In these images normalization to (MNI) space has been performed. Asymmetric decreased putamen and caudate uptake can be seen in the patient with PD. In **(B)** the figure shows; a template region object map overlying: the putamen and caudate nucleus bilaterally in a normalized FDOPA ADD image from a PD patient; the FDOPA template (created from five healthy controls) and the canonical single-subject T1 MRI found in SPM 99. **(C)** Shows the template object map, with volumes shaded, for the ventral striatum and caudate nucleus overlain on the canonical single-subject T1 MRI in sagittal section and next to it a spatially normalized K_I_ image in sagittal section with the ventral striatum and caudate nucleus object map overlain. Also shown is a spatially normalized coronal section K_I_ map through the ventral striatum with the object map overlain.

An ROI analysis was performed using a fixed object map template after stereotaxic transformation of all images into standard (MNI) space. This approach effectively normalizes brain position and shape and so avoids variability of regions due to head size and position and subjective free hand definition. Standard ROI object maps that outlined the left and right hemisphere VS, caudate, and putamen were defined on the MNI single-subject ROI in stereotaxic space (Moore et al., 2003, 2008). The standard object map was placed onto the transformed K_I_ maps, and values for specific FDOPA uptake were obtained for each region (see Figure 1 for ROIs). When performing our ROI analysis a manual correction for head movement was employed as described previously (Whone et al., 2003a).

To limit the number of comparisons, and because of our specific *a priori* hypothesis, *K*_I_ values (units: ml g^−1^ min^−1^) for the caudate and putamen ROIs were averaged to form left and right dorsal striatum (DS) ROIs. Exploratory data analysis revealed that *K*_I_ and NS data were normally distributed, and so we examined the relation between them using Pearson’s correlation coefficient. Statistical significance was set at *p* < 0.01.

## Results

Mean ± SD scores in our PD sample for NS2 (Impulsivity) and NS3 (Extravagance) were 3.1 ± 1.6 and 3.1 ± 1.7, respectively. Whilst population norms for individual NS facets are unavailable, the total NS score (NS1 + NS2 + NS3 + NS4) of the current PD sample (13.6 ± 4) was comparable to that published for age-matched healthy male controls (*n* > 1000; mean age 67 ± 8; total NS = 11.6 ± 5; Stallings et al., 1996). Mean ± SD FDOPA *K*_I_ values for left and right VS and left and right DS are reported in Table 2.

**Table 2:** **FDOPA *K*_I_ values in regions of interest**.

Region	FDOPA *K*_I_ values (mean ± SD)
Left ventral striatum	0.0127 ± 0.0015
Right ventral striatum	0.0120 ± 0.0014
Left dorsal striatum	0.0115 ± 0.0018
Right dorsal striatum	0.0108 ± 0.0015

**Figure 2:**
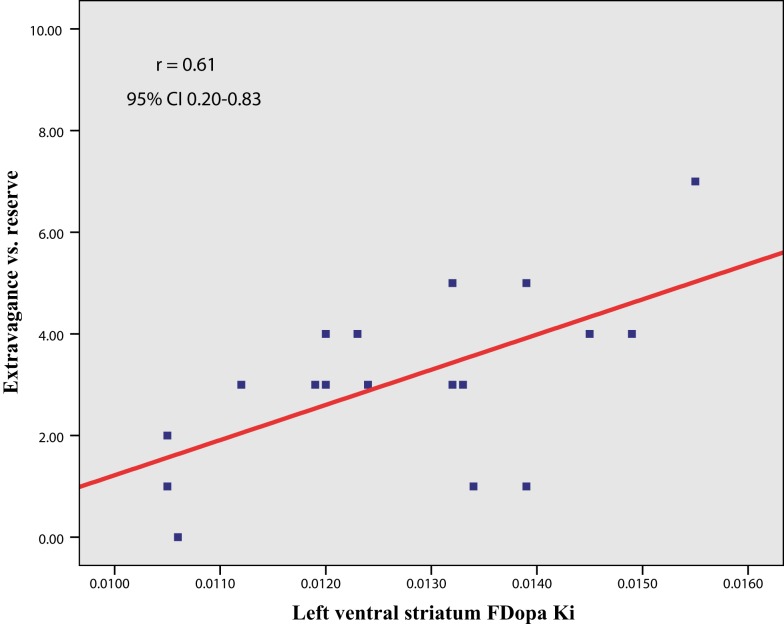
**Scatter plot (and line of best fit) showing the relation between trait disinhibition (NS3, Extravagance vs. reserve) and ventral striatal dopamine synthesis capacity (FDOPA *K*_I_) in Parkinson’s disease**.

In line with our *a priori* hypothesis, we found a significant correlation between NS3 (Extravagance) scores and left VS FDOPA *K*_I_ values [*r* (16) = 0.61, *p* = 0.008, 95% CI 0.20 – 0.83; Figure 2]. This relationship was unchanged when controlling for both age and GDS depression scores (*r* = 0.63, *p* = 0.009). There was a similar trend in the right VS that failed to reach our pre-specified level of statistical significance (*r* = 0.53, *p* = 0.026). (An additional analysis revealed that the slightly greater correlation between left VS FDOPA *K*_I_ and NS3 was not a result of asymmetry of Parkinsonian symptoms). Furthermore, neither left (*r* = −0.13, *p* = 0.60) nor right (*r* = −0.22, *p* = 0.38) VS FDOPA *K*_I_ values correlated with UPDRS motor scores. There was no significant relation between DS FDOPA *K*_I_ values and NS3 scores, although the correlation with right DS values trended toward significance (left, *r* = 0.27, *p* = 0.27; right, *r* = 0.41, *p* = 0.09). Importantly, however, the correlation between left VS FDOPA *K*_I_ values and NS3 scores was significantly greater than that between either left [*t* = 2.59, *p* (1-tail) = 0.01] or right [*t* = 1.46, *p* (1-tail) = 0.08] DS FDOPA *K*_I_ values and NS3 scores.

Against our hypothesis, however, there was no significant correlation between NS2 (Impulsivity) scores and VS FDOPA *K*_I_ values (left, *r* = 0.08, *p* = 0.77; right, *r* = 0.30, *p* = 0.22). Nor was there a significant correlation between NS2 scores and DS *K*_I_ values (left, *r* = −0.12, *p* = 0.65; right, *r* = 0.22, *p* = 0.37). The correlation between left VS FDOPA *K*_I_ values and NS3 (Extravagance) scores was significantly greater than that between left VS FDOPA *K*_I_ values and NS2 (Impulsivity) scores [*t* = 2.67, *p* (1-tail) = 0.008].

## Discussion

Our findings provide new insights into the neurobiology of potential vulnerability to impulse control problems in PD. Consistent with our hypothesis, we found that variation in trait disinhibition (vs. constraint), a continuously varying endophenotype for EXT or impulse-control psychopathology, was associated with levels of DA synthesis capacity in the VS. Individuals with higher levels of trait disinhibition, in particular, tendencies to financial irresponsibility and extravagance, had greater DA synthesis capacity, as indexed by FDOPA *K*_I_ values in the left (and to a lesser extent, right) ventral, but not dorsal, striatum.

Although ICDs in PD have to date been treated as categorical (present/absent) disorders, it is now widely accepted that such disorders do not delineate highly discrete and easily distinguished categories. Rather, they delineate continuous, normally distributed underlying propensities to experience psychopathology, with personality traits linked to behavioral disinhibition forming the core of an EXT spectrum (Clark, 2005; Krueger et al., 2007). I.e., psychopathology represents the extremes of continuously distributed temperament traits. This general temperamental propensity to EXT, rather than its manifestations in particular disorders is therefore a key focus for etiological investigations.

Whilst a recent study linked genetic variation in DDC activity to an EXT endophenotype (sensation seeking) in healthy individuals (Derringer et al., 2010), and sensation seeking has been linked to increased ventral striatal BOLD-fMRI response during reward anticipation (Bjork et al., 2008; see also Yau et al., 2012), ours is the first demonstration that variation in trait disinhibition is linked to variation in DDC activity in the VS in PD, an important brain region underlying inter-individual variation in behavioral disinhibition in preclinical models (Dalley et al., 2011).

We found that only the r-NS subscale NS3 (Extravagance vs. Reserve) was related to ventral striatal DA synthesis capacity. There was no such relation with the NS2 subscale (Impulsivity vs. Reflection), despite the relatively high correlation between NS2 and NS3 (*r* = 0.45). Both subscales showed a similar range of variation in our PD sample, suggesting this is not the reason for a lack of correlation between NS2 scores and VS FDOPA *K*_I_ values. NS3 appears more trait-like (evidenced by much higher test-rest correlation) than NS2 (Takeuchi et al., 2011), and appears to index those traits (irresponsibility, inability to approach life planfully) most strongly linked to general EXT risk (Krueger and South, 2009). It is particularly notable that only NS3 correlates with impaired decision-making on the Iowa Gambling Task (Álvarez-Moya et al., 2011), which has been linked to increased VS FDOPA *K*_I_ values (Schlagenhauf et al., 2012).

On first glance, our results appear inconsistent with an earlier study that failed to find a correlation between trait NS and VS FDOPA *K*_I_ values in unmedicated male PD patients (Kaasinen et al., 2001). However, that study used the total NS score from the original NS scales described by Cloninger (1987), and it is now clear (Ando et al., 2004) that the genetic and environmental factor structure of NS does not resemble the phenotypic structure of NS as originally proposed, making interpretation of that previous null result difficult.

FDOPA is not a specific ligand for DA neurons but rather is trapped by all neurons that contain DDC (Brown et al., 1999). Hence, it is a marker for all tissues that take up and store monoamines, including serotonin (5-HT) as well as DA neurons (Tison et al., 1991). 5-HT has also been implicated in various aspects of impulsivity (Winstanley et al., 2005; Miyazaki et al., 2011). However, *post hoc* analysis of a midbrain raphe ROI (Moore et al., 2008), a structure in which FDOPA is a validated marker of serotonergic function in PD (Pavese et al., 2012), in our sample, found no significant correlation between FDOPA *K*_I_ values in the raphe and either NS2 (Impulsivity) or NS3 (Extravagance) scores (*r* = 0.11, *p* = 0.67 and *r* = 0.32, *p* = 0.2, respectively). Further, in MPTP treated monkeys, Karimi et al. (2013) found a near perfect correlation (*n* = 16, *r*^2^ = 0.95) between striatal FDOPA *K*_I_ values obtained *in vivo* with PET and post-mortem striatal DA levels (measured using high performance liquid chromatography with electrochemical detection). It is likely, therefore, that individual differences in trait disinhibition are primarily related to individual differences in DA synthesis capacity.

This interpretation is consistent with several other recent findings on the neurobiology of impulse-control and addiction vulnerability. D2 autoreceptors located on DA nerve terminals exert a negative feedback regulation that reduces DA synthesis, DA neuron firing, and DA release (Wolf and Roth, 1990; Zhu et al., 1992). Bello et al. (2011) generated mice deficient in such D2 autoreceptors. These “autoDrd2KO” mice displayed elevated striatal DA synthesis and strikingly, demonstrated increased sensitivity to the rewarding and psychomotor stimulant properties of cocaine, together with a greatly exaggerated motivation to work for food reward. I.e., heightened DA synthesis in the autoDrd2KO mice resulted in an addiction-prone, disinhibited phenotype. In humans, striatal DA synthesis capacity measured with [β-^11^C]DOPA PET is negatively correlated with striatal D2 receptor density measured with [^11^C]raclopride PET (Ito et al., 2011). Given that many pre-synaptic D2 receptors in the striatum are DA autoreceptors (Sesack et al., 1994), increased DA synthesis capacity in humans may similarly reflect reduced D2 autoreceptor function. Notably, in animal models, variation in trait impulsivity, measured by premature responding on a 5-choice serial reaction time test of visual attention, has been linked to relatively reduced D2 receptor levels in the VS, functioning as autoreceptors (Besson et al., 2010).

Consistent with this, in humans, Buckholtz et al. (2010) (see also Zald et al., 2008) recently found that trait non-planning impulsivity was inversely correlated with D2/D3 autoreceptor availability in the substantia nigra/ventral tegmental area, measured using [18F]fallypride PET. Furthermore, Van Leare et al. (2009) found a selective negative correlation between cannabinoid CB1 receptor density, measured using [18F]MK-9470 PET, in left hemisphere limbic regions and NS3 (Extravagance) scores in healthy volunteers. Since CB1 receptor activation causes inhibition of DA synthesis (Moranta et al., 2004), reduced density of CB1 receptors would presumably result in higher levels of DA synthesis, and the negative correlation between CB1 receptor density and NS3 is consistent with the positive correlation between ventral striatal DA synthesis capacity and NS3 seen here.

Furthermore, Bello et al. (2011) found that autoDrd2KO mice showed exaggerated phasic DA release, resulting from a larger releasable pool of DA generated by the lack of DA synthesis inhibition in DA terminals. Similarly, in both monkeys (Doudet and Holden, 2003) and human individuals with PD (Piccini et al., 2003), the magnitude of amphetamine-induced striatal DA release as measured by [11C]-raclopride PET (an indirect measure of phasic DA release, Grace, 2008), is positively correlated with DA synthesis capacity measured using FDOPA PET. In humans, Buckholtz et al. (2010) found that trait disinhibition predicted greater amph-induced DA release (together with stronger drug-primed wanting) and the relation between midbrain autoreceptor availability and trait impulsivity was mediated via this enhanced amphetamine-induced striatal DA release.

The influence of variation in DA synthesis capacity on EXT tendency might result from DA’s established role in reward processing, particularly the attribution of incentive salience (Berridge, 2007). Incentive salience is a motivational component of reward, one that transforms sensory information about rewards and reward cues into attractive, “wanted” incentives, motivating pursuit (Berridge, 2007). Notably, in animals, there is considerable individual variability in incentive salience attribution, and rats with a strong propensity to attribute incentive salience to reward cues also show heightened behavioral disinhibition, risk-taking, and an increased tendency to seek drugs like cocaine (Flagel et al., 2010). Available data suggest that animals prone to attribute incentive salience to reward cues have a more active DA system than those who do not (Marinelli and White, 2000; Tomie et al., 2000). In healthy volunteers VS FDopa *K*_I_ values have been found to positively correlate with BOLD-fMRI activity to reward cues in brain regions linked to incentive salience attribution (Siessmeier et al., 2006), and ventral striatal BOLD-fMRI response to reward cues in humans is increased as a function of trait non-planning impulsivity and is linked to genetic variation in the D2 (auto)-receptor (Forbes et al., 2009).

Of particular relevance to the development of extreme impulse control problems following treatment with dopaminergic medication in PD, rats with heightened incentive salience attribution show a greater propensity for dopaminergic drug-induced sensitization – a form of neuroplasticity hypothesized to play a key role in addiction (Flagel et al., 2008). In particular, the incentive-sensitization theory (Robinson and Berridge, 2008) posits that excessive drug use arises from the excessive attribution of incentive salience to drug rewards and their cues, due to progressive neuroadaptations in DA projections to ventral striatal motivation circuitry. Strikingly, PD individuals with DA dysregulation syndrome were shown to exhibit potentiated l-DOPA-induced ventral striatal DA release, which correlated with exaggerated drug-primed l-DOPA wanting, together with enhanced responding to monetary reward (Evans et al., 2006). Also, PD (and non-PD) individuals with disordered gambling showed impaired DA D2 autoreceptor activity (Ray et al., 2012) and exaggerated VS DA release to gambling and other reward cues (O’Sullivan et al., 2011; see also Steeves et al., 2009; Joutsa et al., 2012a), consistent with incentive-sensitization.

It is possible that, rather than reflecting an underlying neurobiological risk factor for ICDs in PD, increased VS FDOPA uptake could be the result of disease-related and/or drug-induced neuroplastic changes leading to disease- and/or drug-induced disinhibition. In the current sample of early-stage PD patients, who had only recently begun DA replacement therapy, however, mean ventral striatal FDOPA *K*_I_ values were very similar to those of healthy controls (Whone et al., 2004) and were unrelated to severity of PD. In animal models, there is some evidence that repeated treatments with DA agonist drugs can enhance basal DA synthesis in the VS (Rowlett et al., 1997) and frontal cortex (Chernoloz et al., 2012). However, other studies show persistent inhibition of DA synthesis following repeated treatment with DA agonists (Imperato et al., 1996). A study of detoxified alcoholics found no differences in VS DA synthesis capacity measured with FDOPA PET (Heinz et al., 2005). Very recently, Joutsa et al. (2012b) found increased medial orbitofrontal, but not ventral striatal, FDOPA uptake in 10 individuals with manifest ICDs in PD. Since disinhibitory traits were not measured in the patients or controls of that study, however, it may be that several of the controls had high EXT risk, explaining the lack of difference in VS FDOPA uptake between ICD cases and controls. Frontal, but not striatal, DA synthesis is also increased following stress (Reinhard et al., 1982), suggesting that enhanced frontal, but not ventral striatal, FDOPA uptake in ICD cases may reflect state, as opposed to trait, effects.

Furthermore, Giladi et al. (2007) and Bastiaens et al. (2013) found that the appearance of heightened impulse control problems in affected PD individuals began only after a median interval of ∼2 years of treatment, and our patients had only recently been started on dopaminergic medication, within the previous 4–12 weeks, and were on only low doses of either l-DOPA or DA agonist. In addition, Voon et al. (2007) found that NS scores were not influenced by dopaminergic therapy. Importantly, numerous studies have found a shared genetic diathesis to underlie associations between EXT disorders and traits related to disinhibition, including NS (Young et al., 2000; Agrawal et al., 2004). Most critically, Derringer et al. (2010) found that genetic variation in DDC activity predicted variation in trait disinhibition, suggesting that variation in DDC activity reflects a trait EXT vulnerability, rather than a disease or medication-related effect (see also Engeln et al., 2012). Likewise, Flagel et al. (2010), found that, in rats, variation in incentive salience attribution and behavioral disinhibition are genetically influenced, correlated traits that depend on ventral striatal DA for their expression (Saunders and Robinson, 2012).

Hence, we think it highly unlikely that our findings of a positive correlation between levels of self-reported disinhibition and VS DA synthesis capacity are a result of PD and its treatment with dopaminergic medication. Rather, we think they reflect genetically influenced, pre-existing neurobiological individual differences in VS DA synthesis capacity in PD patients, underpinning variation in trait disinhibition or EXT propensity, which in turn is associated with future ICD morbidity risk (as PD advances and dopaminergic drug therapy increases), resulting from an increased susceptibility to DAergic-drug-induced incentive-sensitization (Boileau et al., 2006). Longitudinal studies will be required to establish the validity of this account.

In conclusion, we have shown that temperamental vulnerability to impulse control or EXT problems such as disordered gambling in male PD patients is related to relatively greater DA synthesis capacity in the ventral, but not dorsal, striatum. Our results are consistent with preclinical models of EXT risk, and may prove informative in understanding the psychological and neurobiological mechanisms whereby individual differences in temperament contribute to the development of impulse control and addictive pathologies in the context of the treatment of PD and other neurological disorders.

## Conflict of Interest Statement

The authors declare that the research was conducted in the absence of any commercial or financial relationships that could be construed as a potential conflict of interest.
